# Exploiting the Photoresponse in LiInP_2_Se_6_ for Image Processing

**DOI:** 10.1021/acs.nanolett.5c05227

**Published:** 2026-04-06

**Authors:** Anshul Rasyotra, Anirban Chowdhury, Dipanjan Sen, Subir Ghosh, Arpan Ghosh, Rui Gusmao, Divya Somvanshi, Joan M. Redwing, Zdenek Sofer, Saptarshi Das

**Affiliations:** † Engineering Science and Mechanics, 201943 The Pennsylvania State University, University Park, Pennsylvania 16802, United States; ‡ Department of Inorganic Chemistry, University of Chemistry and Technology Prague, 160 00 Prague, Czech Republic; § Department of Physics, 92991 Harcourt Butler Technical University, Kanpur 208002, India; ∥ 2DCC, The Pennsylvania State University, University Park, Pennsylvania 16802, United States; ⊥ Materials Science and Engineering, The Pennsylvania State University, University Park, Pennsylvania 16802, United States; # Electrical Engineering, The Pennsylvania State University, University Park, Pennsylvania 16802, United States

**Keywords:** 2D materials, thiophosphates, field effect transistors, anomalous photoresponse, negative photoconductivity, image processing

## Abstract

We report an anomalous photoresponse in LiInP_2_Se_6_-gated monolayer MoS_2_ field effect transistors (FETs), driven by sub-bandgap photocarrier excitation and relaxation in LiInP_2_Se_6_. The MoS_2_/LiInP_2_Se_6_ heterostructure exhibits gate-tunable persistent negative photoconductivity, a rare phenomenon in 2D FET platforms. Notably, the photoconductivity change scales with incident light intensity, with weaker illumination producing slower, smaller responses and stronger illumination inducing faster, stronger suppression. Exploiting the nonlinear, intensity-dependent photoresponse of LiInP_2_Se_6_, we demonstrate an image-processing platform that enables contrast modulation directly at the sensor level. By varying two controllable parameters, namely, applied top-gate bias and light exposure time, the device response can be tuned to modify the contrast of the image. This intrinsic behavior illustrates how contrast tunability can be achieved on a chip, offering a simple and compact route toward elementary preprocessing functions in vision devices.

## Graphical abstract

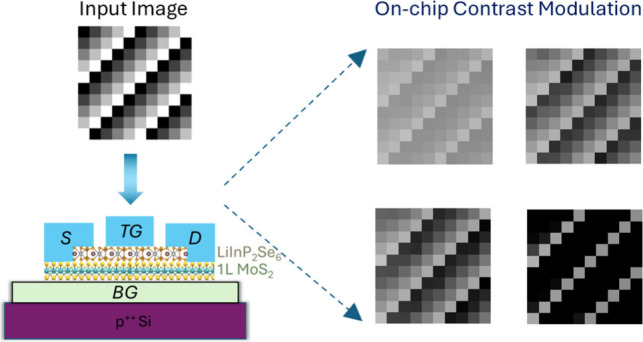

Achieving compact, energy-efficient vision systems that mimic the adaptability and dynamic range of biological perception is a major goal for neuromorphic hardware.^1,2^ Conventional image sensors^3–5^ rely on complex peripheral circuitry to handle variable lighting conditions and enhance contrast, which increases the system footprint and power consumption. A promising alternative is to embed adaptive sensing and preprocessing directly into the material platform,^5–8^ enabling in-sensor control of signal transduction in response to environmental stimuli.

Layered semiconductors and insulators, particularly van der Waals (vdW) materials,^9–13^ offer a rich landscape of band alignments and interfacial charge transfer behaviors that can be harnessed for such functionality. However, despite this versatility, most 2D material-based optoelectronic devices primarily rely on positive photoconductive responses, while negative photoconductive effects remain far less explored. To date, negative photoconductance phenomena have been predominantly investigated in thin films^14,15^ and nanowires,^16–18^ with only a limited number of reports demonstrating these responses in atomically thin materials. For example, Xie et al. observed the negative photoconductance behavior in Cr_2_Ge_2_Te_6_ photodetectors arising from trap-mediated carrier dynamics, with a transition from negative photoconductance to positive photoconductance as the excitation power was increased. Similarly, wavelength- or power-dependent switching between negative photoconductance and positive photoconductance has been reported in FePS_3_ and InAs nanosheet photodetectors, attributed to hot-carrier trapping and excitation-dependent transport processes.^19,20^

In addition to intrinsic electronic effects, environmental adsorption and enhanced carrier-phonon or carrier-carrier scattering, potentially driven by photothermoelectric or photobolometric mechanisms, have also been suggested to contribute to negative photoconductance behavior in 2D systems.

Among these, LiInP_2_Se_6_,^21,22^ a bimetallic thiophosphate, exhibits an unusual, rarely reported sub-bandgap photoresponse. Photoluminescence measurements reveal the presence of midgap states that enable sub-bandgap absorption, and subsequent charge separation that leads to trapping of photoexcited electrons within LiInP_2_Se_6_, while photogenerated holes are transferred to an adjacent MoS_2_ channel in a vdW heterostructure device. This interfacial charge transfer results in a persistent and intensity-dependent suppression of MoS_2_ conductivity. We observe that the rate and the change in magnitude of photoconductivity are directly tied to the incident light intensity. Lower intensities result in slower, shallower responses, while higher intensities produce a faster, deeper suppression of channel conductance. Notably, the photoconductivity remains negative and persistent, unlike typical optoelectronic field effect transistors (FETs) based on 2D semiconductors^23–28^ that show positive persistent photoconductivity.^29–31^

This nonlinear, light intensity-dependent negative photoresponse is leveraged to implement image processing using adaptive contrast modulation without the need for external control or processing units. The resulting architecture is inherently compact with all key functionality emerging from the intrinsic optoelectronic behavior of the LiInP_2_Se_6_/MoS_2_ heterostructure. Our findings establish LiInP_2_Se_6_ as a unique optoelectronic material facilitating image processing and provide new insights into interfacial photophysics in layered semiconductors/dielectrics with defect-mediated charge transfer.

LiInP_2_Se_6_ crystals, synthesized through a chemical vapor transport (CVT) route, are layered van der Waals thiophosphates that crystallize in a trigonal structure (space group *P*3̅1*c*). Each individual layer in this crystal framework is composed of ethane-like [P_2_Se_6_]^4–^ anions, which contain formally P^4+^ cations coordinated with Se^2–^ anions. Within this unit, two P^4+^ centers are linked by a P–P bond, forming a [P–P]^4+^ dimer that is further surrounded by six selenide ions. Moreover, the stacking of these individual layers follows an ABAB sequence in which the InSe_6_ polyhedra are aligned in successive layers, while the LiSe_6_ and P_2_Se_6_ polyhedra alternate periodically along the *c*-axis. This bonding arrangement gives rise to a highly ordered and structurally stable layered lattice characteristic of LiInP_2_Se_6_. Its clean cleavage planes and robust stacking order make LiInP_2_Se_6_ particularly well-suited for integration into 2D heterostructures and gated electronic devices. To assess the structural and optical quality of LiInP_2_Se_6_, we employed a suite of characterization techniques. The top and side view schematics of the LiInP_2_Se_6_ crystal structure are shown in Figure 1a. Additionally, the optical micrograph of the exfoliated crystal presented in Supporting Information Figure 1 confirm that LiInP_2_Se_6_ can be mechanically exfoliated into thin, uniform flakes exceeding 20 μm in lateral size, conducive to high-yield device fabrication. The atomic force microscopy (AFM) image of the flake shown in Figure 1b confirms low surface roughness and high uniformity. Scanning electron microscopy (SEM) and energy-dispersive X-ray spectroscopy (EDX) images shown in Figure 1c distinctly verify the presence of indium, phosphorus, and selenium, aligning well with the expected InP_2_Se_6_ framework of LiInP_2_Se_6_. Note that Li cannot be directly detected by EDS due to its low atomic number. Moreover, the X-ray diffraction (XRD) pattern of the LiInP_2_Se_6_ crystal exhibits sharp and intense peaks corresponding to the (002), (004), (006), and (008) planes as shown in Figure 1d. This dominant out-of-plane diffraction pattern indicates a strong crystallographic orientation along the *c*-axis, suggesting that LiInP_2_Se_6_ is highly layered and crystalline.

**Figure 1:**
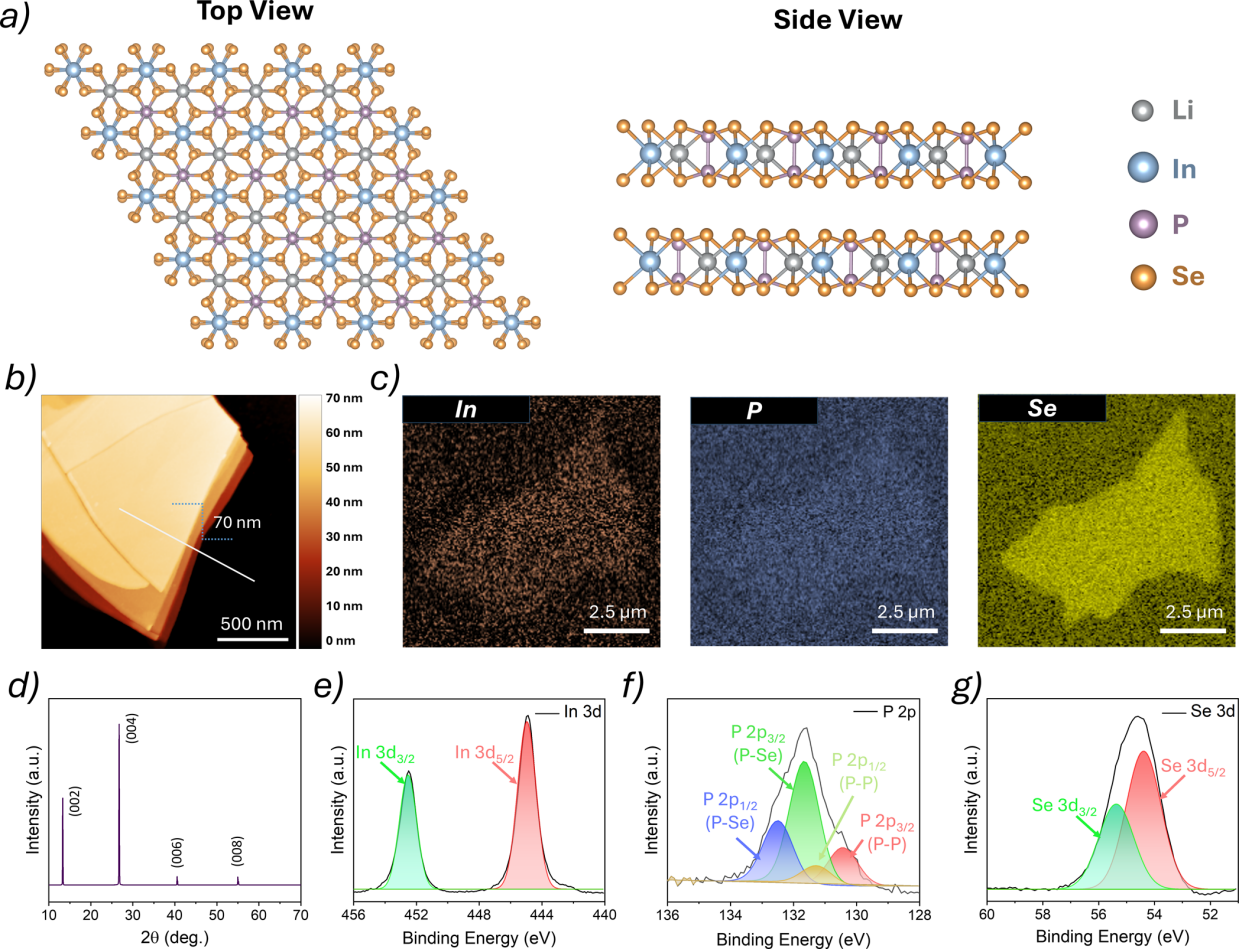
Structural and compositional characterization of LiInP_2_Se_6_. (a) Top and side views of the LiInP_2_Se_6_ crystal structure. (b) Atomic force microscopy (AFM) image of an exfoliated LiInP_2_Se_6_ flake with a measured thickness of ∼70 nm. (c) Scanning electron microscopy (SEM) combined with energy-dispersive X-ray spectroscopy (EDS) verifies the presence of LiInP_2_Se_6_ constituents, except Li, which cannot be detected by EDS. (d) X-ray diffraction (XRD) pattern of LiInP_2_Se_6_ crystals that confirms phase purity with no secondary phases. (e–g) X-ray photoelectron spectroscopy (XPS) spectra of exfoliated LiInP_2_Se_6_, showing the core level peaks corresponding to the In 3d, P 2p, and Se 3d orbitals, respectively.

Next, X-ray photoelectron spectroscopy (XPS) was carried out to analyze the elemental composition and chemical states in LiInP_2_Se_6_. As shown in Figure 1e–g, the In 3d core level spectrum reveals a dominant In 3d_5/2_ peak at 445.26 eV, characteristic of In^3+^ in a selenide environment. The P 2p region exhibits two doublets, reflecting distinct bonding environments within the [P_2_Se_6_]^4–^ anion, with each doublet having the 2p_3/2_:2p_1/2_ constraints (Δ ∼ 0.87 eV, 2:1 area ratio, identical FWHM). The lower BE doublet originates from P–P bonding, while the higher-BE doublet corresponds to P–Se coordination. Structural stoichiometry indicates P–P 1:3 P–Se bonding ratio, also reflected in the proportional integrated areas of corresponding deconvoluted components. The Se 3d peak appears at 54.50 eV, consistent with a selenide chemical state. Due to the proximity of the Se 3d and Li 1s binding energies, the Li signal could not be distinctly resolved.

Furthermore, in the following sections, we present the integration of LiInP_2_Se_6_ as a top-gate dielectric in MoS_2_ FETs. This vdW integration ensures a clean, trap-free interface and provides enhanced electrostatic control. Beyond its dielectric functionality, LiInP_2_Se_6_ exhibits defect-mediated sub-bandgap optical absorption,^32^ which plays a key role in shaping the optoelectronic behavior of the 2D FETs. This absorption mechanism gives rise to persistent, intensity-dependent negative photoconductivity in the MoS_2_ channel, enabling hardware-integrated image-processing functionalities in the LiInP_2_Se_6_-gated 2D FET architecture.

To evaluate the electrostatic strength of LiInP_2_Se_6_ as a top-gate dielectric, we fabricated dual-gated MoS_2_ FETs by integrating exfoliated LiInP_2_Se_6_ flakes on top of monolayer MoS_2_ channels. The device architecture, shown schematically in Figure 2a and imaged by SEM in Figure 2b, consists of a 25 nm Al_2_O_3_ back-gate dielectric deposited via atomic layer deposition (ALD) over a Ti/Pt gate electrode, and a top-gate stack formed by transferring LiInP_2_Se_6_ flakes followed by patterned Ni/Au top-gate contacts. Ni/Au source and drain electrodes contact the MoS_2_ channel, forming a vertical vdW heterostructure.

**Figure 2:**
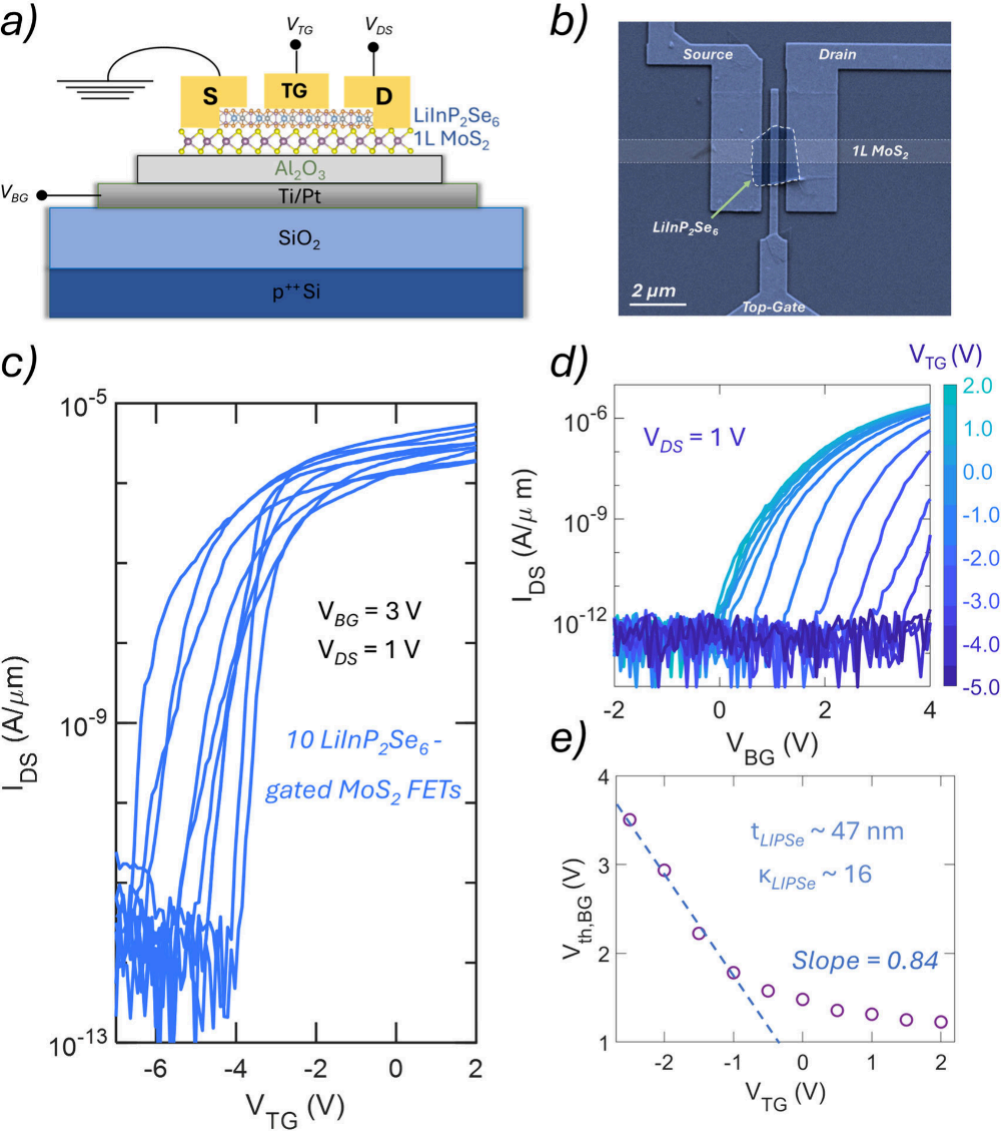
LiInP_2_Se_6_ as a top-gate dielectric for monolayer MoS_2_ FETs. (a) Schematic and (b) false-colored angled scanning electron microscopy (SEM) image of a dual-gated 2D FET incorporating LiInP_2_Se_6_ as the top-gate dielectric, monolayer MoS_2_ as the semiconducting channel, and 25 nm Al_2_O_3_ as the back-gate dielectric. (c) Top-gate transfer characteristic of 10 MoS_2_ FETs gated with LiInP_2_Se_6_ obtained by sweeping the *V*_TG_ from −7 to 2 V at a constant *V*_BG_ of 3 V and a constant *V*_DS_ of 1 V. (d) Back-gate transfer characteristics, i.e., source to drain current (*I*_DS_) measured by sweeping the back-gate voltage (*V*_BG_) for different top-gate voltages (*V*_TG_) at a constant source-to-drain voltage (*V*_DS_ = 1 V) and e) back-gate threshold voltage (*V*_th,BG_) extracted using the iso-current method at an *I*_DS_ of 10 nA/μm as a function of *V*_TG_ for LiInP_2_Se_6_-gated MoS_2_ FETs. The slope of the curve of *V*_th,BG_ versus *V*_TG_ is proportional to the ratio of the back-gate and top-gate EOT values. The effective dielectric constant was found to be ∼16 for a 47 nm thick LiInP_2_Se_6_ flake.

Supporting Information Figure 2 shows the back-gate transfer characteristics, i.e., source-to-drain current (*I*_DS_) measured by sweeping the back-gate voltage (*V*_BG_) at a constant source-to-drain voltage (*V*_DS_ = 1 V) of five representative devices without applying the top-gate bias, confirming standard monolayer MoS_2_ n-type behavior.^33–36^ Figure 2c shows the top-gate transfer characteristics, i.e., *I*_DS_ as a function of the top-gate voltage (*V*_TG_) at a constant *V*_BG_ of 3 V for 10 MoS_2_ FETs gated with LiInP_2_Se_6_, demonstrating high on/off current ratios, steep subthreshold swings (SS), etc. To further assess the electrostatic modulation capability, we measured the top-gate transfer characteristics at various *V*_BG_ values as shown in Supporting Information Figure 3, confirming strong electrostatic modulation via LiInP_2_Se_6_. Note that in a dual-gated 2D FET architecture (Figure 2b), the back gate modulates the full MoS_2_ channel, including the channel under the source and drain contacts, while the top gate modulates only a part of the channel region between contacts. *V*_BG_ primarily influences charge injection, whereas *V*_TG_ controls channel depletion. To quantify the electrostatic control of the top-gate dielectric, we measured back-gate transfer characteristics at different fixed *V*_TG_ values as shown in Figure 2d and extracted the corresponding threshold voltages (*V*_th,BG_) using an iso-current method at an *I*_DS_ of 10 nA/μm. The extracted *V*_th,BG_ values plotted against *V*_TG_ (Figure 2e) exhibit a linear relationship in the depletion regime, and the slope yields the ratio of back-gate to top-gate capacitance (*C*_BG_/*C*_TG_).^37^

Using the known *C*_BG_ of ≈3.3 × 10^–3^ F m^–2^ for 25 nm Al_2_O_3_, we estimate *C*_TG_ ≈ 2.8 × 10^–3^  F m^–2^ for the LiInP_2_Se_6_ gate stack. AFM analysis (Supporting Information Figure 4) shows a LiInP_2_Se_6_ thickness of ∼47 nm, yielding a relative dielectric constant κ of ≈16, comparable to conventional high-κ dielectrics. Moreover, the gate leakage currents (*I*_TG_) remained below 10^–3^ A/cm^2^ across the full range of top-gate voltages (Supporting Information Figure 5), confirming the insulating quality and breakdown robustness of the exfoliated LiInP_2_Se_6_ layers.

Together, these results establish LiInP_2_Se_6_ as a high-quality, high-κ vdW gate dielectric capable of providing strong electrostatic control with excellent reliability and low leakage in 2D FETs. We also measured the top-gate transfer characteristics of LiInP_2_Se_6_-gated MoS_2_ FETs over 50 consecutive cycles at three different temperatures (25, 50, and 75 °C) as shown in Supporting Information Figure 6. The data demonstrate excellent cycle-to-cycle reproducibility and thermal stability, with minimal variation in the top-gate threshold voltage across the investigated temperature range, confirming the robustness of the LiInP_2_Se_6_ gating mechanism under repeated electrical stress and increased operating temperatures. This robust platform sets the stage for exploring the distinct sub-bandgap optoelectronic properties of LiInP_2_Se_6_ in the following sections.

To explore the optoelectronic behavior of LiInP_2_Se_6_-gated MoS_2_ FETs, we investigated their photoresponses under controlled illumination conditions. Top-gate transfer characteristics were measured at a fixed *V*_BG_ of 3 V under both dark and illuminated conditions as a function of incident light power (*P*_IN_). As shown in Figure 3a, an increase in *P*_IN_ leads to a systematic rightward shift of the transfer curves, indicating a monotonic increase in the top-gate threshold voltage (*V*_th,TG_). Concurrently, the overall channel conductance decreases with an increase in *P*_IN_, an unusual effect known as negative photoconductivity. This phenomenon is further illustrated in the time-resolved photoresponse shown in Figure 3b, where exposure to a long-lived optical pulse induces a persistent decrease in *I*_DS_. After the cessation of illumination, the current recovers slowly over tens of minutes, a hallmark of persistent photoconductivity. This persistent effect complicates repeated sensing, but we find that the devices can be reliably reset to their preillumination state by applying a single *V*_TG_ sweep at a *V*_BG_ of 3 V as demonstrated in Supporting Information Figure 7.

**Figure 3:**
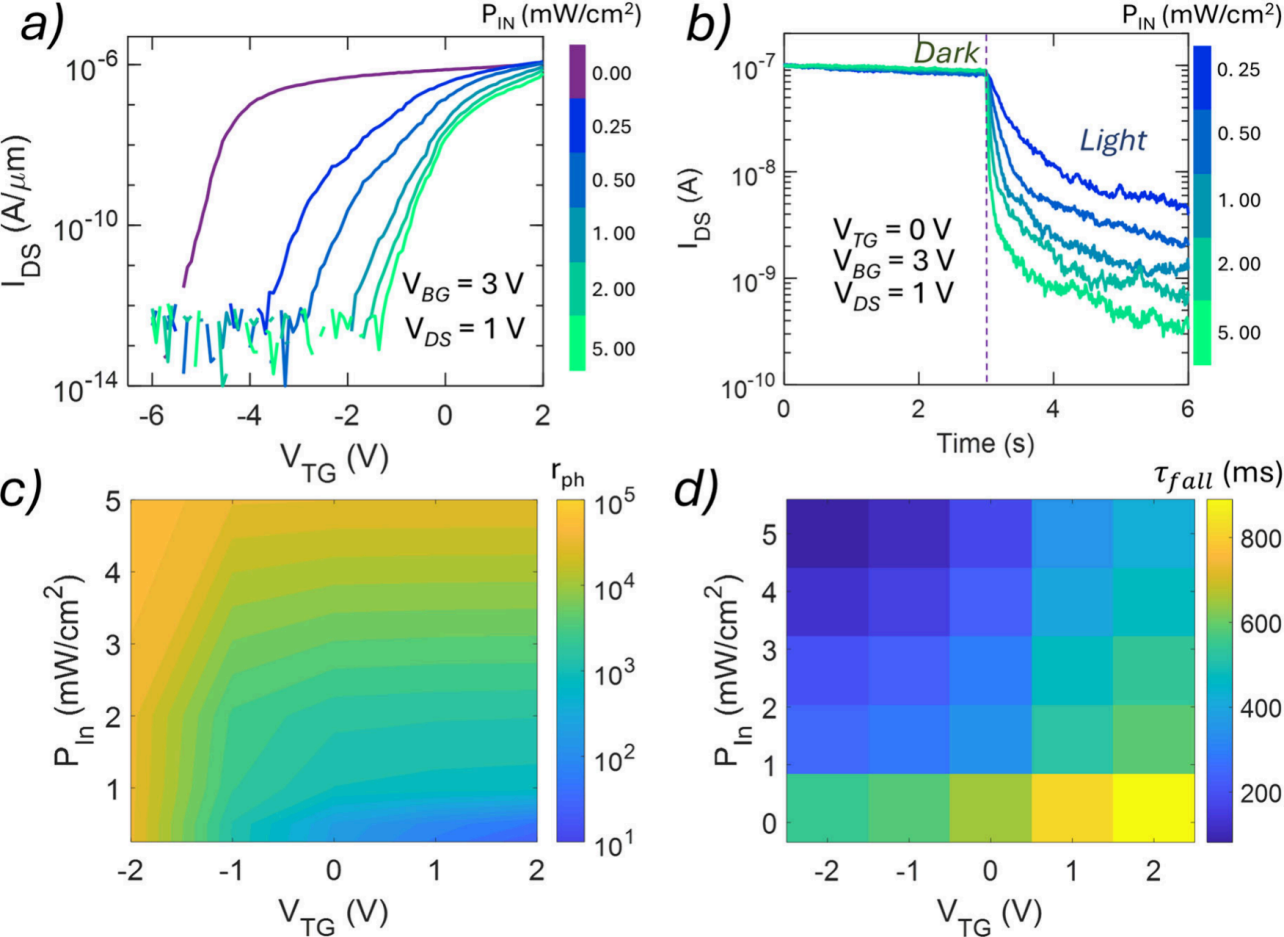
Tunable negative photoconductivity in LiInP_2_Se_6_-gated MoS_2_ FETs. (a) Top-gate transfer characteristics of a representative LiInP_2_Se_6_-gated MoS_2_ FET under dark and different illumination intensities (*P*_IN_ ranging from 0 to 5 mW/cm^2^) at a constant *V*_BG_ of 3 V and a constant *V*_DS_ of 1 V. (b) Time-resolved photoresponse (*I*_DS_ vs time) of a representative 2D FET exhibiting a negative photoresponse at various illumination intensities, revealing a power-dependent dynamic response. The light was kept ON in the time duration of 3–6 s. (c) 2D color map of the extracted photoresponsivity ratio (*r*_ph_) as a function of *P*_IN_ and *V*_TG_, illustrating optimal gate bias for maximizing *r*_ph_. (d) 2D map of the fall time (τ_fall_) extracted from the transient decay profiles, showing that a faster response (lower τ_fall_) is achieved at a more negative *V*_TG_ and a higher *P*_IN_, suggesting favorable switching dynamics.

To identify the underlying mechanism of this unusual negative photoconductivity in LiInP_2_Se_6_-gated MoS_2_ FETs, we performed photoluminescence (PL) measurements over varied temperatures on a LiInP_2_Se_6_ flake as presented in Supporting Information Figure 8, which reveal broad sub-bandgap emission, indicative of midgap defect states capable of capturing photocarriers. The PL spectra of LiInP_2_Se_6_ exhibit a prominent emission peak near ∼1.7 eV at temperatures below 100 K, indicating radiative recombination through defect-associated states. As depicted in ref 32, the PL peak for LiInP_2_Se_6_ is expected around 2.06 eV; however, the observed red-shift in our measurements suggests the influence of intrinsic defects, particularly Li vacancies, which can introduce localized states within the bandgap. Notably at higher temperatures, the PL intensity decreases markedly due to thermally activated nonradiative processes and sub-bandgap, defect-mediated trapping and recombination of photocarriers. This temperature-dependent behavior underscores the role of native defects in shaping the optical properties of LiInP_2_Se_6_.

Additionally, we attribute the negative photoconductivity to a photogating mechanism dominated by electron trapping in LiInP_2_Se_6_. As illustrated in the band diagram schematic (Supporting Information Figure 9), illumination of LiInP_2_Se_6_ generates electron-hole pairs via sub-bandgap absorption mediated by deep defect states near the valence band maxima (VB_m_) of LiInP_2_Se_6_. Photogenerated electrons become trapped in these sub-bandgap states within LiInP_2_Se_6_, creating a localized negative charge near the MoS_2_ channel. Photogenerated holes, meanwhile, are energetically favorable to get transferred into the MoS_2_ valence band. The net result is a reduction in electron density in the MoS_2_ channel, suppressing *I*_DS_ and shifting *V*_th,TG_ positively under illumination.

To further probe the microscopic origin of these states, we performed first-principles density functional theory (DFT) calculations (Supporting Information Figure 9a,b), which shows that Li vacancies in LiInP_2_Se_6_ introduce deep defect levels that lie close to the VB_m_ of MoS_2_. These states serve as efficient electron traps and facilitate interfacial hole transfer. Additionally, we attribute the negative photoconductivity to a photogating mechanism dominated by electron trapping in LiInP_2_Se_6_. As illustrated in the band diagram schematic (Supporting Information Figure 9c,d), illumination of LiInP_2_Se_6_ generates electron-hole pairs via sub-bandgap absorption mediated by deep defect states near the valence band maxima (VB_m_) of LiInP_2_Se_6_. Photogenerated electrons become trapped in these sub-bandgap states within LiInP_2_Se_6_, creating a localized negative charge near the MoS_2_ channel. Photogenerated holes, meanwhile, are energetically favorable to transfer into the MoS_2_ valence band. The net result is a reduction in electron density in the MoS_2_ channel, suppressing *I*_DS_ and shifting *V*_th,TG_ positively under illumination.

Furthermore, we performed controlled experiments on back-gated MoS_2_ FETs without a LiInP_2_Se_6_ top gate. These devices showed a negligible change in conductance under identical illumination (Supporting Information Figure 10), confirming that the observed behavior originates from the LiInP_2_Se_6_ layer and its interface with MoS_2_, rather than intrinsic MoS_2_ photophysics.

Next, the magnitude of the photoconductivity change is strongly dependent on the applied *V*_TG_ during illumination. Supporting Information Figure 11 presents the time-resolved negative photoresponse as a function of *V*_TG_ and *P*_IN_. Figure 3c presents a color map of the photoconductivity ratio (*r*_ph_), defined as the ratio of pre- and postillumination conductivity, as a function of *P*_IN_ and *V*_TG_. At higher values of *P*_IN_, *r*_ph_ exhibits a nonmonotonic dependence on *V*_TG_. This behavior arises from the exponential sensitivity of the MoS_2_ channel conductance to gate-induced threshold shifts near the subthreshold regime. When the device is biased near threshold, even a modest positive shift in *V*_th,TG_ due to illumination results in a significant suppression of channel conductance. In contrast, when biased deep in the ON state, the same *V*_th,TG_ shift produces an only marginal change in conductance. On the other hand, for devices initially biased in the OFF or deep subthreshold regime, the preillumination current is already low, and the postillumination current may fall below the measurement limit, saturating the response. This interplay defines an optimal *V*_TG_ biasing window, where the device exhibits a maximum sensitivity and dynamic range. By tuning *V*_TG_ appropriately, we can achieve a discernible photoconductive response spanning a change in *P*_IN_ of more than 4 orders of magnitude, demonstrating broad response tunability over varying illumination intensities. We also observe that the fall time (τ_fall_) of the photoresponse decreases with an increase in *P*_IN_, indicating that the rate of conductance suppression also encodes the incident light intensity. Figure 3d shows a color map of τ_fall_ as a function of *P*_IN_ and *V*_TG_. This intensity-dependent kinetics, combined with persistent and reversible photoconductivity, enables analog optical encoding directly at the device level, without the need for external amplifiers or postprocessing circuitry as we will demonstrate below.

To demonstrate the functional advantage of the nonlinear, intensity-dependent photoresponse in LiInP_2_Se_6_-gated MoS_2_ FETs, we implemented a hardware level adaptive contrast enhancement scheme using a 10  × 10 pixel HDR image as shown in Figure 4a. The unique kinetics of these devices, where higher light intensities induce faster and stronger conductance suppression while lower intensities yield slower and weaker responses, naturally lend themselves to contrast enhancement without the need for external processing.

**Figure 4:**
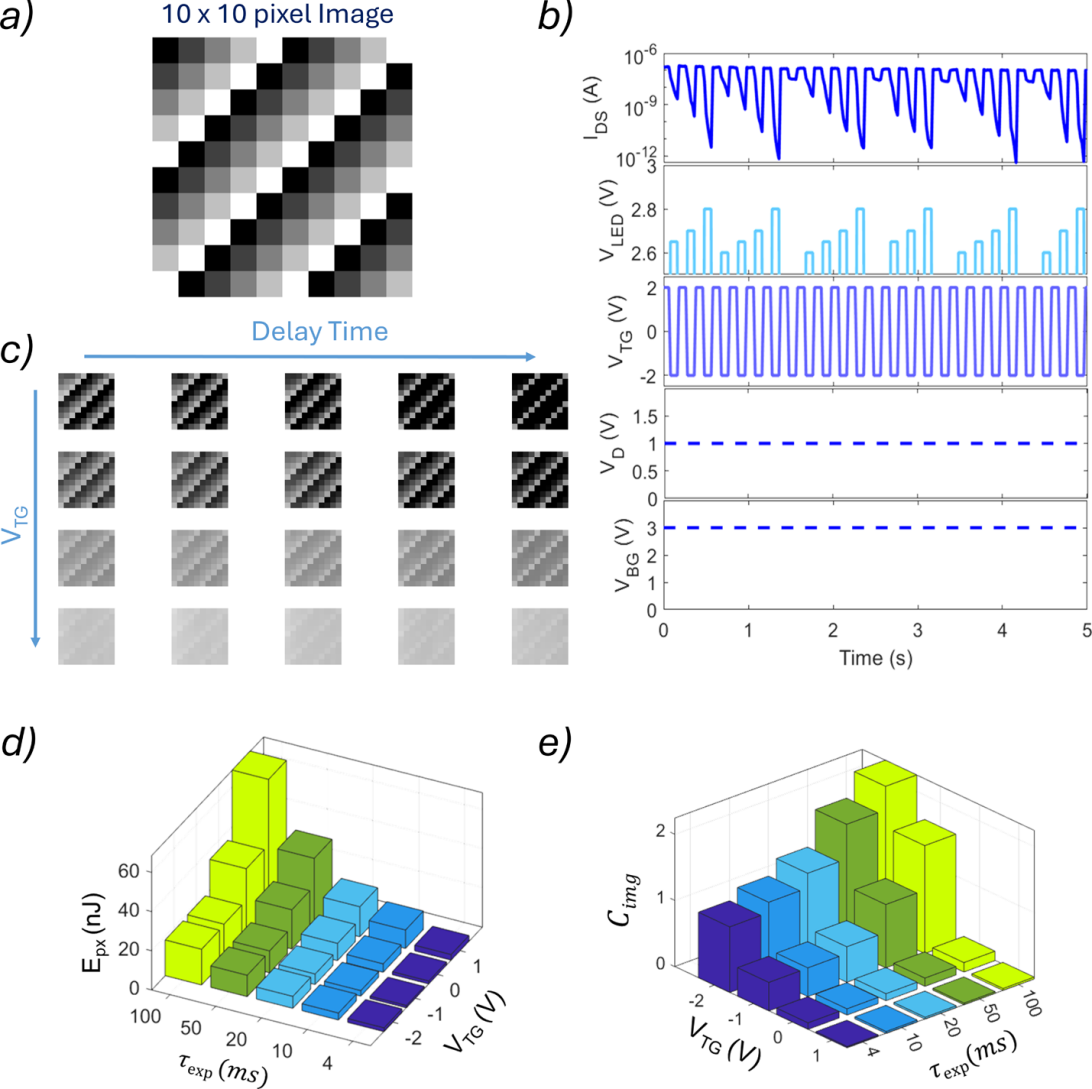
Adaptive image contrast enhancement via a nonlinear, intensity-dependent photoresponse in LiInP_2_Se_6_-gated MoS_2_ FETs. (a) A 10 pixel × 10 pixel test image used to evaluate hardware level contrast enhancement. (b) Measurement protocol. During each light exposure, a fixed top-gate voltage (*V*_TG_) is applied to generate a photocurrent (*I*_D_). The device is subsequently reset to its dark state using a *V*_TG_ pulse of 2 V. *V*_BG_ and *V*_DS_ remain constant at 3 and 1 V, respectively, throughout the measurement. (c) Resulting conductance maps for varying *V*_TG_ (rows) and delay time (columns) values. A short delay time and a positive *V*_TG_ yield nearly linear mapping (minimal contrast change), whereas a longer delay time and a negative *V*_TG_ strongly suppress high-intensity pixels, enhancing the image contrast. (d) Energy consumed per pixel and (e) normalized contrast index (*C*_img_) showing a pronounced increase in contrast for a more negative *V*_TG_ and a longer delay time as the device enters its nonlinear response regime.

In our experimental setup, each image pixel is mapped to the corresponding *P*_IN_. The device is then exposed to light for a controlled duration (τ_exp_), during which a fixed top-gate voltage (*V*_TG_) is applied to regulate the sensitivity. After exposure, the devices are returned to the dark state, and a reset pulse (*V*_TG_ = 2 V) is applied to restore the initial conductance, ensuring reusability. *V*_BG_ and *V*_DS_ are kept constant throughout, and the full voltage protocol is illustrated in Figure 4b. After exposure, the resulting conductance maps for different *V*_TG_ and τ_exp_ conditions are shown in Figure 4c. We find that shorter τ_exp_ and more positive *V*_TG_ result in a nearly linear mapping of intensity to conductance, preserving the original grayscale contrast. In contrast, a longer τ_exp_ and a more negative *V*_TG_ dramatically enhance contrast, as the dynamic range of the photoresponse becomes more nonlinear, strongly suppressing high-intensity pixels while preserving lower-intensity ones. This adaptive transformation of the image contrast is achieved intrinsically through device physics without any external amplification or computation.

To quantify contrast enhancement, we define a simple contrast index (*C*_img_) based on the standard deviation of pixel conductance values normalized by their mean:

$$C_{\mathrm{img}}=\sigma_{\mathrm{img}}/\mu_{\mathrm{img}}$$

where σ_img_ and μ_img_ are the standard deviation and mean of the postillumination pixel conductance values, respectively. As shown in Figure 4d, *C*_img_ increases significantly with a more negative *V*_TG_ and a longer τ_exp_, peaking when the nonlinear regime of the photoresponse is fully activated. In parallel, we estimate the energy consumed per pixel during image acquisition and encoding. As shown in Figure 4e, the energy expenditure remains in the nanojoule range per pixel (∼1 nJ), even at long τ_exp_ and high *V*_TG_ values, owing to the ultralow operating currents and compact active area (<1 μm^2^). This highlights the ultra-low-power nature of the proposed vision system.

In summary, we report an unconventional, intensity-dependent negative photoconductivity in MoS_2_ FETs gated with layered thiophosphate dielectric LiInP_2_Se_6_. This behavior originates from sub-bandgap absorption in LiInP_2_Se_6_ and subsequent defect-mediated charge transfer, where photogenerated electrons become trapped in LiInP_2_Se_6_ and holes are transferred to the MoS_2_ channel, leading to a persistent suppression of conductance. The magnitude and kinetics of this response are highly tunable via top-gate bias and illumination intensity, enabling precise control over both signal amplitude and encoding speed. We exploit this nonlinear and programmable photoresponse to construct an image-processing platform capable of adaptive contrast modulation. By simply adjusting the light exposure time and gate bias, the device can operate in either linear or enhanced contrast mode, all while consuming subnanojoule energy per pixel and requiring no peripheral processing. These findings demonstrate that defect-engineered van der Waals dielectrics can introduce distinct optoelectronic functionalities into 2D FETs and open opportunities for low-power visual processing approaches. Together, these results position LiInP_2_Se_6_ not only as a high-κ gate dielectric but also as a light-responsive material that can contribute to sensing and signal-processing tasks within future device platforms.

## Supplementary Material



## Data Availability

Data sets generated and/or analyzed during the current study and the codes used for plotting the data are available from the corresponding authors upon request.

## Notes

Optical image of a LiInP_2_Se_6_ crystal, electrical characteristics of back-gated and top-gated MoS_2_ FETs, AFM thickness measurements, gate leakage assessments, stability and endurance studies over repeated gate sweeps, recovery dynamics, low-temperature PL, DFT-based defect analyses, gate-tunable photoresponse data, and methods section, which includes all of the experimental details (PDF)
