# Supplementary material for: Exploiting the Photoresponse in LiInP2Se6 for Image Processing
Source: Nano Lett. 2026 Apr 6;26(15):4951–8. doi: 10.1021/acs.nanolett.5c05227 (PMC13107444; doi:10.1021/acs.nanolett.5c05227)
Supplement: Supplementary file 1 [file nl5c05227_si_001.pdf]

Supporting Information

Exploiting the Photoresponse in $LiInP_2Se_6$ for Image Processing

Anshul Rasyotra[1], Anirban Chowdhury[1], Dipanjan Sen[1], Subir Ghosh[1], Arpan Ghosh[1], Rui Gusmao[2], Divya Somvanshi[3], Joan M Redwing[4,5], Zdenek Sofer[2], and Saptarshi Das[1,5,6]*

[1]Engineering Science and Mechanics, Penn State University, University Park, PA 16802, USA

[2] Dept. of Inorganic Chemistry, University of Chemistry and Technology Prague, Prague 166 28, Czech Republic

[3]Department of Physics, Harcourt Butler Technical University, Kanpur, India

[4]2DCC, Penn State University, University Park, PA 16802, USA

[5]Materials Science and Engineering, Penn State University, University Park, PA 16802, USA

[6]Electrical Engineering, Penn State University, University Park, PA 16802, USA

[*] Corresponding Author

Supporting Information 1

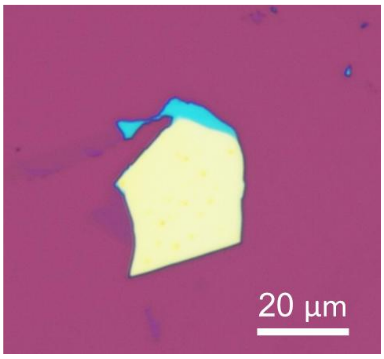

Supporting Information Figure 1. Optical image of a $LiInP_2Se_6$ crystal. *Optical micrograph of an exfoliated $LiInP_2Se_6$ flake exhibiting a lateral size ~ 25µm.*

Supporting Information 2

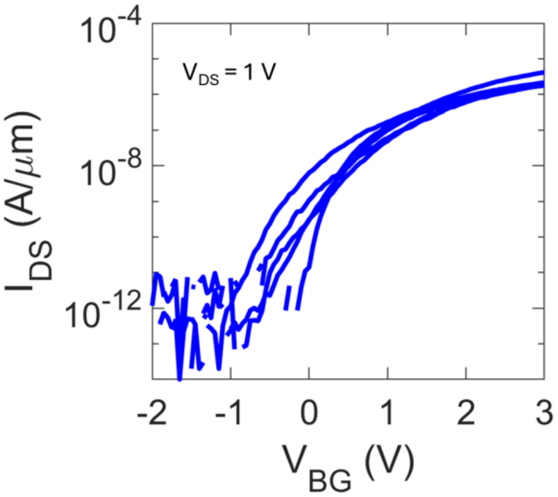

Supporting Information Figure 2. Back-gated MoS_2 FET. *Back-gate transfer characteristics of 5 MoS_2 FETs were assessed by sweeping the V_{BG} from 3 V to -2 V for V_{DS} =1 V.*

Supporting Information 3

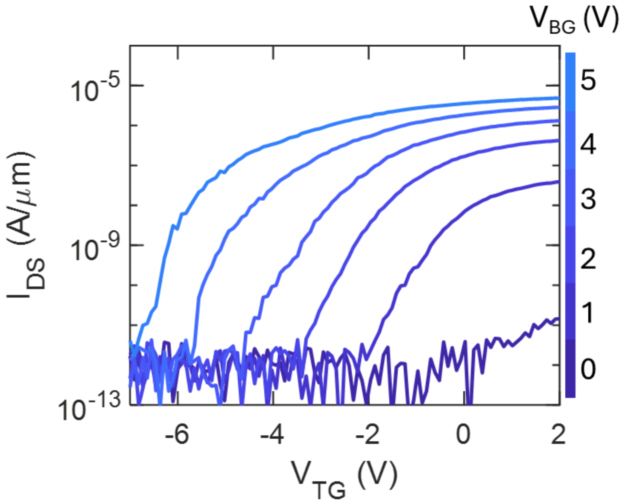

Supporting Information Figure 3. $LiInP_2Se_6$-gated MoS_2 FET. *Top-gate transfer characteristics of a representative MoS_2 FET gated with $LiInP_2Se_6$ was measured by sweeping the V_{TG} from -7 V to 2 V for various V_{BG} values at a constant V_{DS} =1 V.*

Supporting Information 4

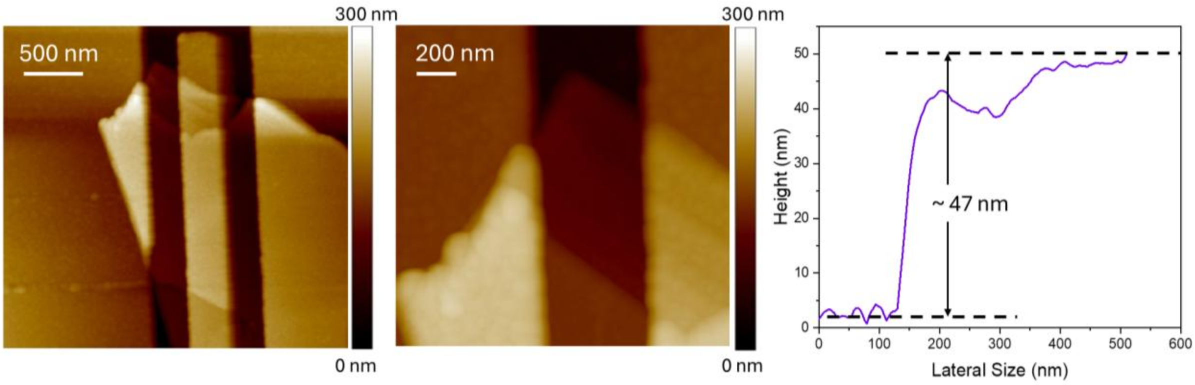

Supporting Information Figure 4. Atomic force microscopy (AFM) image of $LiInP_2Se_6$ as a top-gate dielectric. *AFM image of an exfoliated $LiInP_2Se_6$ flake used as a top-gate dielectric for MoS_2 FET, with a measured thickness of ~47 nm as highlighted by the corresponding line scan.*

Supporting Information 5

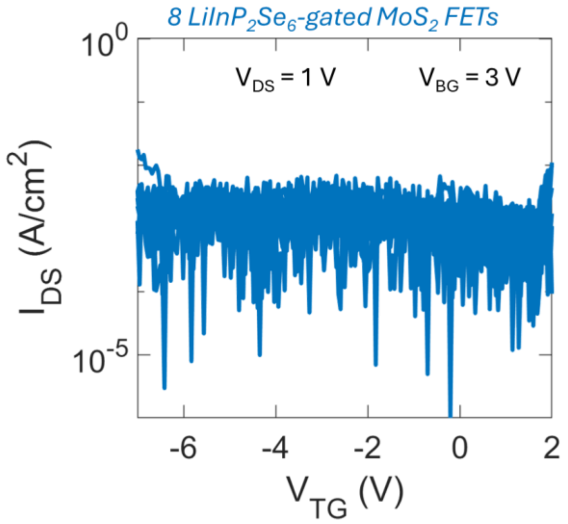

Supporting Information Figure 5. Gate leakage current of $LiInP_2Se_6$-gated MoS_2 FET. *Gate leakage current of 8 representative MoS_2 FETs gated with $LiInP_2Se_6$ was measured by sweeping the V_{TG} from -7 V to 2V at a constant V_{BG} = 3V and a constant V_{DS} =1 V.*

Supporting Information 6

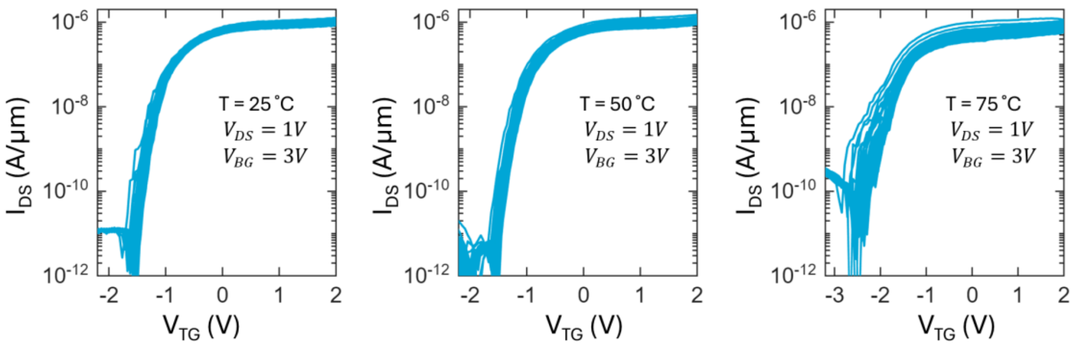

Supporting Information Figure 6. Temperature-dependent operational stability of $LiInP_2Se_6$-gated MoS_2 FETs. *Top-gate transfer characteristics showing the drain current I_{DS} as a function of top-gate voltage V_{TG}, measured over 50 consecutive gate-voltage sweeps at 25°C, 50°C and 75°C, with a fixed drain bias of $V_{DS} = 1V$. The minimal cycle-to-cycle variation and negligible threshold-voltage shift across the investigated temperature range demonstrate the excellent thermal and operational stability of the $LiInP_2Se_6$-gated MoS_2 FETs under repeated electrical stressing.*

Supporting Information 7

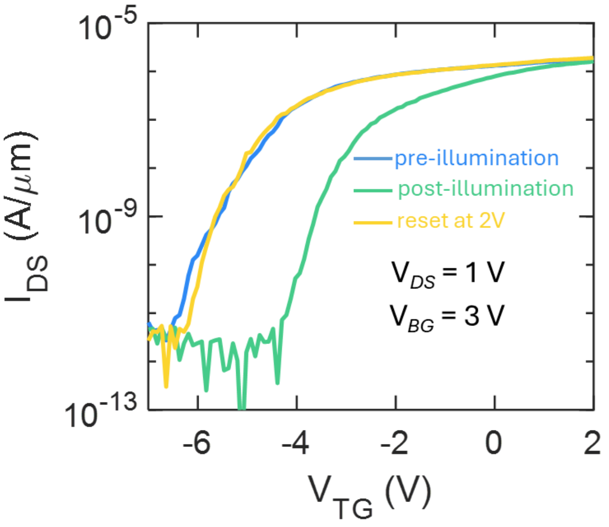

Supporting Information Figure 7. Resetting of $LiInP_2Se_6$-gated MoS_2 FET. *Top-gate transfer characteristics of a representative $LiInP_2Se_6$-gated MoS_2 FET measured before illumination, after illumination, and after resetting the device by applying V_{TG}= 2 V. Characteristics were*

Supporting Information 8

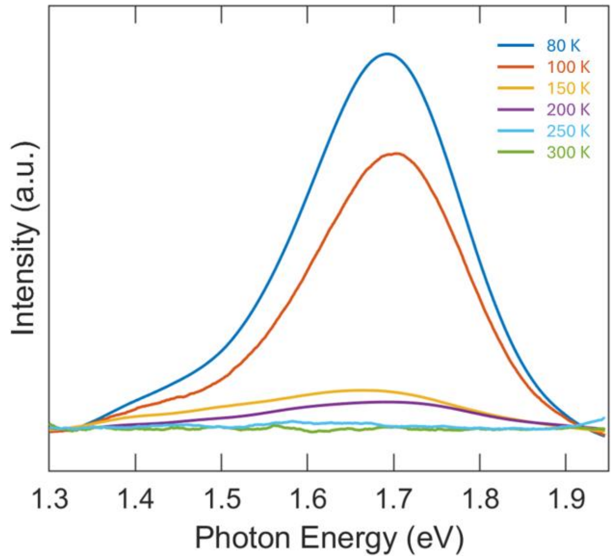

Supporting Information Figure 8. Temperature-dependent PL spectra of $LiInP_2Se_6$ flakes (80-200 K). *The increased PL intensity and redshift observed in $LiInP_2Se_6$ at lower temperatures suggest defect-induced localized states, likely arising from Se and/or Li vacancies.*

Supporting Information 9

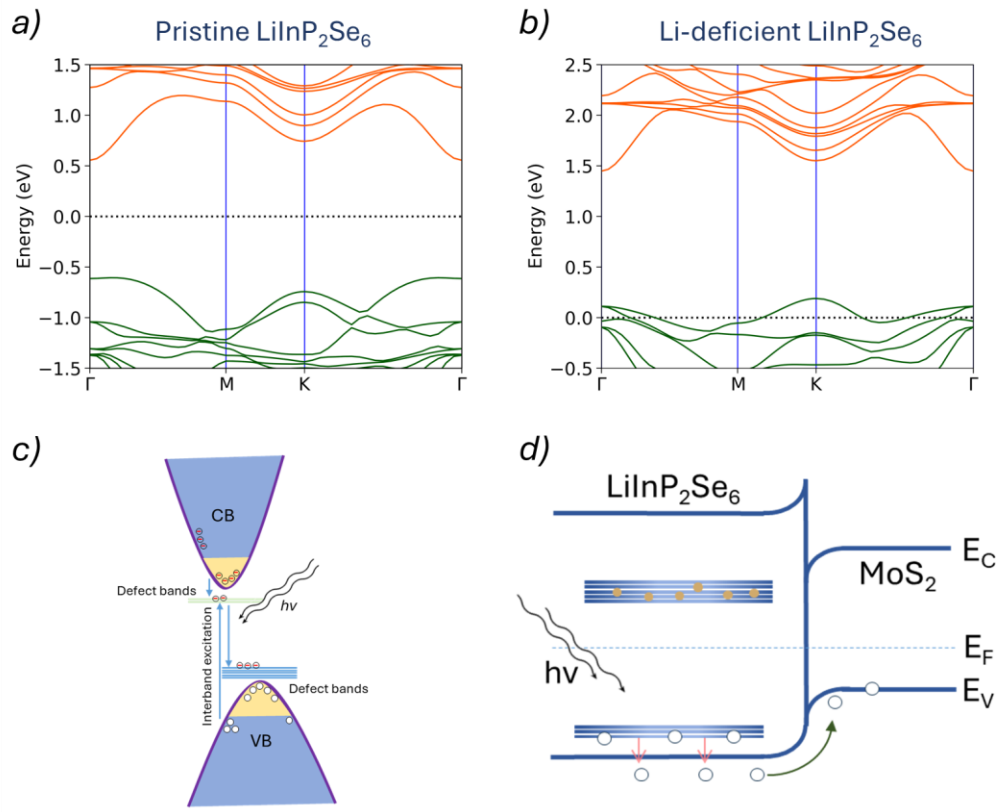

Supporting Information Figure 9. Defect-mediated interfacial charge transfer in $LiInP_2Se_6/MoS_2$ heterostructure. *a) Density functional theory (DFT) calculations illustrating the electronic band structures of a pristine $LiInP_2Se_6$ and b) a Li-deficient $LiInP_2Se_6$, highlighting the effect of Li vacancies on the interfacial charge transfer. c) Schematic illustration of photocarrier excitation and relaxation mechanism in $LiInP_2Se_6$, indicating the role of defect bands in the photoresponse. d) Schematic band diagram illustrating sub-bandgap photoexcitation in $LiInP_2Se_6$ through deep defect states near the valence band maxima followed by transfer of photogenerated holes into the MoS_2 valence band.*

Supporting Information 10

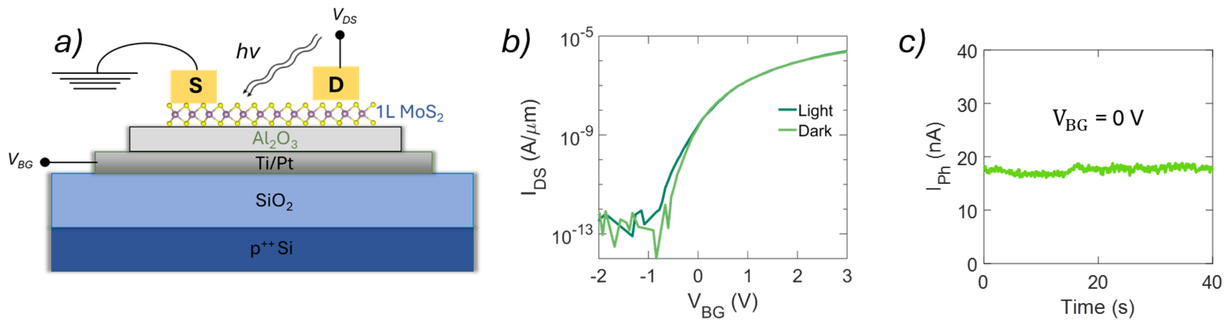

Extended Data Figure 8. Photoresponse in back gated MoS_2 FET. *a) Schematic of a back-gated 2D FET. b) Back-gate transfer characteristics, i.e., source to drain current (I_{DS}) measured by sweeping the back-gate voltage (V_{BG}) under dark and light illumination. c) Time-resolved photoresponse (I_{DS} vs. time) of a representative back-gated MoS_2 FET displaying negligible photoresponse under identical illumination.*

Supporting Information 11

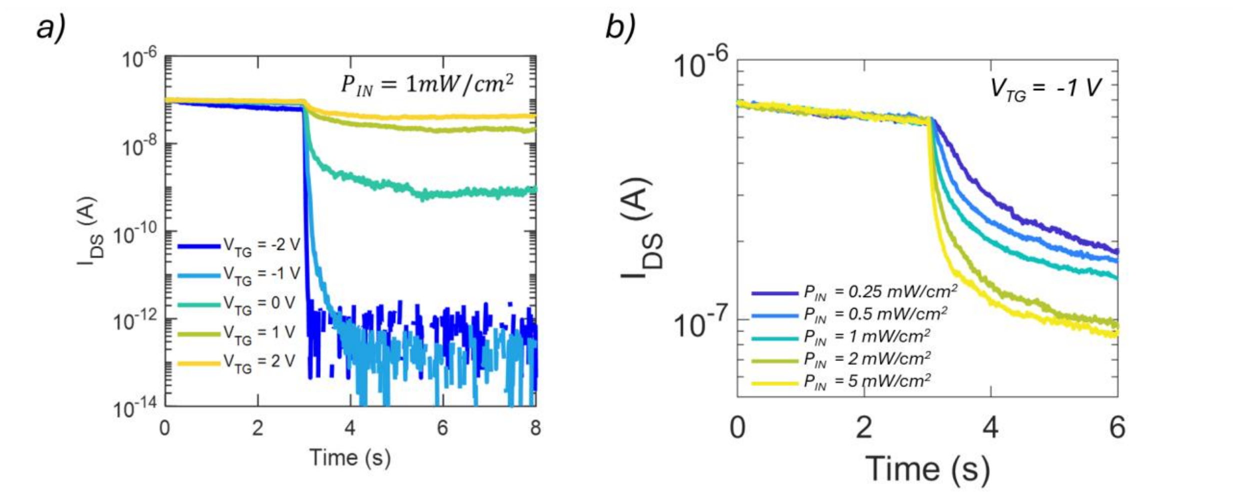

Supporting Information Figure 11. Gate tunable dynamic photoresponse in $LiInP_2Se_6$-gated MoS_2 FET. *Time-resolved negative photoresponse under constant illumination (P_{IN} = 1 mW/cm²) at **(a)** varying V_{TG} and **(b)** varying illumination at constant V_{TG}. An increase in the magnitude and speed of the negative photoresponse is observed with increasing negative gate bias.*

Methods

Synthesis of $LiInP_2Se_6$ Single Crystals.

$LiInP_2Se_6$ single crystals were grown by chemical vapor transport (CVT) in a two-zone horizontal furnace. Approximately 2.5 g of pre-synthesized polycrystalline $LiInP_2Se_6$ and 50 mg of iodine (I_2) as the transport agent were sealed under vacuum in a fused silica ampoule (inner diameter ≈ 16 mm, outer diameter ≈ 18 mm, length ≈ 28 cm) using an oxy-natural gas torch. The sealed tube was placed with the source and growth zones maintained at 660 °C and 560 °C, respectively. The source zone temperature was raised from ambient to 560 °C over 12 h, held for 4 h, further increased to 660 °C within 3 h, and kept constant for 144 h before cooling to room temperature in 12 h. The deposition zone followed a similar profile: ramped to 660 °C in 12 h, held for 4 h, lowered to 560 °C within 3 h, maintained for 144 h, and cooled over 12 h. Crystals collected from the deposition zone inside a N_2-filled glovebox exhibited smooth, uniform surfaces and were used directly for device fabrication due to their high moisture sensitivity.

Growth of Monolayer MoS_2

Monolayer MoS_2 films were synthesized by metal-organic chemical vapor deposition (MOCVD) on pre-scored, double-side-polished c-plane sapphire substrates (2 in.). Growth was performed in a cold-wall horizontal reactor with an inductively heated graphite susceptor and wafer rotation to ensure uniformity. Molybdenum hexacarbonyl ($Mo(CO)_6$) and hydrogen sulfide (H_2S) served as precursors, with $Mo(CO)_6$ maintained at 10 °C and 950 Torr in a stainless-steel bubbler (0.036 sccm flow) and H_2S introduced at 400 sccm. The substrate was pre-annealed at 1000 °C in H_2 for 10 min. Growth proceeded at 1000 °C and 50 Torr for 18 min under a hydrogen atmosphere. After deposition, samples were cooled to 300 °C in H_2S to prevent film decomposition. Detailed growth conditions have been described elsewhere [1-3].

X-ray Diffraction

Powder X-ray diffraction (XRD) was carried out on a Rigaku Miniflex600 diffractometer using Cu Kα radiation (λ = 1.5406 Å) generated at 40 kV and 15 mA. A graphite monochromator and Kβ foil filter were employed, and samples were mounted on zero-background silicon substrates.

X-ray Photoelectron Spectroscopy

X-ray photoelectron spectroscopy (XPS) was performed at room temperature on a Thermo Scientific NEXSA G2 system equipped with an electron flood gun and a scanning ion gun. Samples were adhered to copper tape for mounting, and data were analyzed using Thermo Avantage software.

Atomic Force Microscopy

Atomic force microscopy (AFM) was used to examine the thickness of exfoliated multilayer flakes before and after plasma treatment. Measurements were conducted on a Bruker Dimension Icon instrument with RTESPA-150 probes in peak-force tapping mode (12 nN peak force, 0.5 Hz scan rate). Image analysis and data extraction were performed using Gwyddion software.

Scanning Electron Microscopy/ Energy-Dispersive X-ray Spectroscopy

Top-gated MoS_2 transistor structures were imaged using a Zeiss Gemini 500 field-emission scanning electron microscope operated at 5 kV. Energy-dispersive X-ray spectroscopy (EDS) was performed on micrometer-sized bulk $LiInP_2Se_6$ flakes using an ESEM Q250 microscope with a tungsten source at 30 kV to assess elemental composition.

Photoluminescence Spectroscopy (PL)

Photoluminescence (PL) spectra were recorded using a Horiba LabRAM HR Evolution confocal Raman microscope equipped with a 532 nm laser (34 mW, filtered to 1%). A 100×

objective lens with a numerical aperture of 0.9 and a grating of 300 gr mm^{-1} were used for the measurements.

Device Fabrication

Monolayer MoS_2 was transferred onto 25 nm Al_2O_3 substrates and spin-coated with PMMA A6 (4000 rpm, 45 s), followed by baking at 180 °C for 90 s. Electron-beam lithography (Raith EBPG5200) and development in MIBK:IPA (1:1, 60 s) defined the patterns, which were etched by SF_6 reactive ion etching (RIE) at 5 °C for 30 s. Residual resist was removed by sequential acetone and IPA cleaning. Mechanically exfoliated 2D dielectric flakes were then transferred onto pre-patterned MoS_2 channels. A second lithography step defined source, drain, and top-gate electrodes. MMA EL6 and PMMA A3 resists were spin-coated (4000 rpm, 45 s) and baked at 150 °C and 180 °C for 90 s each. After development, 50 nm Ni/30 nm Au contacts were deposited by electron-beam evaporation. Lift-off was performed in acetone (1 h) followed by IPA rinsing (30 min).

Electrical Characterization

Device measurements were performed under ambient conditions using a semi-automated FormFactor 12000 probe station integrated with a Keysight B1500A parameter analyzer. A continuous-wave white light source was used for photoresponse measurements unless otherwise specified.

Computational Details: The DFT calculations was performed using the linear combination of atomic orbitals (LCAO) basis set with PBE-GGA as an exchange-correlation functional implemented in Quantum Atomistic tool kit X-2025.06 [1] . The LCAO describes the electronic structure using norm-conserving ‘Pseudodojo’ pseudopotential with considerable accuracy and lower computational cost. Brillouin zone integration was performed over Monkhorst-pack grid sampling of 9×9×1 with sufficiently large energy cut-off of 125 Hartree. To avoid any

interaction between the periodic images of the neighboring images, a vacuum region of 20 Å is included in the out-of-plane z- direction. The geometries and lattice parameters of $LiInP_2Se_6$ were fully relaxed employing the LBFGS method with force tolerance less than 0.01 eV/Å and stress tolerance 0.1 Gpa. Spin polarization was not incorporated in this study, as it falls beyond the scope of the present work. The electronic band structure was calculated along high symmetry points of hexagonal lattice system i.e. Γ-M-K-Γ. We used Fermi-Dirac occupation methods for the calculation of Fermi energy (E_F) with respect to vacuum level. A super cell of size 2×2×1 was created, to study the Li vacancy in $LiInP_2Se_6$ monolayer.

References

(1) Sebastian, A.; Pendurthi, R.; Choudhury, T. H.; Redwing, J. M.; Das, S. Benchmarking monolayer MoS2 and WS2 field-effect transistors. *Nature communications* **2021**, *12* (1), 693.

(2) Dodda, A.; Oberoi, A.; Sebastian, A.; Choudhury, T. H.; Redwing, J. M.; Das, S. Stochastic resonance in MoS2 photodetector. *Nature communications* **2020**, *11* (1), 4406.

(3) Ravichandran, H.; Sen, D.; Wali, A.; Schranghamer, T. F.; Trainor, N.; Redwing, J. M.; Ray, B.; Das, S. A Peripheral-Free True Random Number Generator Based on Integrated Circuits Enabled by Atomically Thin Two-Dimensional Materials. *ACS nano* **2023**, *17* (17), 16817-16826.
